# Associations of nutrition knowledge with dietary preferences, symptom burden, and quality of life in women with polycystic ovary syndrome

**DOI:** 10.3389/fnut.2026.1799113

**Published:** 2026-06-29

**Authors:** Bahar Yalçın Çeliktaş, Eslem Altıntaş, Melike Nur Çelikayar, Sinem Dinmez

**Affiliations:** 1 Department of Nutrition and Dietetics, Faculty of Health Sciences, Fenerbahçe University, Istanbul, Türkiye; 2 Department of Nursing, Faculty of Health Sciences, Acibadem Mehmet Ali Aydinlar University, Istanbul, Türkiye; 3 Department of Nutrition and Dietetics, Faculty of Health Sciences, Gazi University, Ankara, Türkiye; 4 Department of Midwifery, Faculty of Health Sciences, Rumeli University, Istanbul, Türkiye

**Keywords:** health-related quality of life, lifestyle and behavior, nutrition knowledge, nutrition literacy, polycystic ovary syndrome

## Abstract

Polycystic ovary syndrome (PCOS) is a complex endocrine disorder in which lifestyle-related factors are closely linked to symptom burden and quality of life. Although nutrition knowledge is frequently emphasized in PCOS management, its association with symptom severity and quality of life remains unclear. This web-based analytical cross-sectional study examined associations between nutrition knowledge, diet-related preferences, lifestyle behaviors, symptom burden, and quality of life among 330 Turkish women aged 18–45 years with a self-reported physician diagnosis of PCOS. Nutrition knowledge and diet-related preferences were assessed using validated instruments, quality of life was assessed using the SF-12, and symptom burden was summarized using an index derived from a PCOS-related symptom checklist. Higher body mass index was associated with greater symptom burden and poorer physical health, whereas regular physical activity was associated with better physical functioning. Nutrition knowledge was positively associated with healthier diet-related preferences but was not associated with symptom burden or quality of life. In multivariable analyses, body mass index remained the strongest predictor of symptom burden, while nutrition knowledge and diet-related preferences were not significant contributors. These findings indicate a knowledge–outcome disconnect in PCOS, suggesting that higher nutrition knowledge may align with healthier preferences without corresponding improvements in health outcomes. Because dietary intake and eating behaviors were not directly measured, conclusions regarding behavior should be interpreted cautiously. Public health and women’s health interventions may benefit from integrating nutrition education with behavior change support, weight management, and physical activity promotion.

## Introduction

Polycystic ovary syndrome (PCOS) is a heterogeneous endocrine disorder affecting approximately 10–15% of reproductive-aged women worldwide. This disorder exerts multifaceted effects on both reproductive and metabolic health (1). The core biological features of the condition include chronic anovulation, hyper-androgenism, polycystic ovarian morphology, insulin resistance, and abdominal obesity, and common clinical manifestations such as hirsutism, acne, difficulty managing weight, and menstrual irregularities adversely affect both physical and psychological well-being (2, 3). Consequently, PCOS is increasingly recognized not only as a hormonal disorder but also as a public health concern influencing quality of life (QoL), psychosocial well-being, and long-term metabolic risk (4).

In addition to genetic susceptibility, environmental and lifestyle-related factors play a decisive role in the pathophysiology of PCOS. Obesity, sedentary behavior, and exposure to endocrine-disrupting chemicals contribute to disease onset and progression through mechanisms involving insulin resistance and chronic low-grade inflammation (5). Considering this multifactorial etiology, international guidelines regard lifestyle modification—particularly balanced nutrition, weight management, and regular physical activity—as the first-line approach in PCOS management (6, 7). Supporting evidence shows that lifestyle-induced weight loss improves insulin resistance, reduces androgen levels, and leads to notable improvements in menstrual irregularity, hirsutism, and metabolic symptoms (8, 9).

Nutrition is a fundamental component of PCOS management. It influences metabolic, hormonal, and behavioral outcomes. However, existing literature indicates that many women with PCOS have inadequate nutrition knowledge, and increased knowledge does not consistently translate into healthier dietary behavior. For example, university students with PCOS have been shown to exhibit low nutrition knowledge scores, which were unrelated to diet quality (10). Even when knowledge improves, behavior change is often inconsistent, demonstrating a pronounced knowledge–behavior gap (11). Emotional eating, body image concerns, weight-management difficulties, stress, environmental influences, and financial constraints further widen this gap (12, 13). Emotional eating tendencies, body dissatisfaction, and disordered eating behaviors have also been shown to adversely affect dietary practices among women with PCOS (14, 15).

Emerging evidence demonstrates that health-related quality of life (HRQoL) is significantly reduced in women with PCOS due to symptom burden, metabolic disturbances, and psychosocial strain, with HRQoL declining significantly as symptom severity increases (16, 17). In contrast, the impact of nutrition knowledge on HRQoL remains inconsistent. Several studies suggest that nutrition knowledge has limited influence on HRQoL and does not independently translate into improved well-being (18). This is consistent with contemporary evidence showing that HRQoL in PCOS is driven primarily by symptom severity, weight status, and psychological distress rather than knowledge alone (19).

To our knowledge, the literature has largely examined these constructs separately, and studies evaluating nutrition knowledge, diet-related preferences, symptom burden, and HRQoL together within a single analytical framework in women with PCOS remain limited (20, 21). Moreover, previous studies have focused on individual components such as dietary intake, eating behaviors, or HRQoL, without integrating symptom burden and lifestyle-related factors, and their interactions remain unclear (22, 23). In this context, the present study aimed to evaluate nutrition knowledge among women with PCOS, to investigate its associations with diet-related preferences, lifestyle behaviors, symptom burden, and HRQoL, and to explore the clinical and public health relevance of the knowledge–behavior gap. This approach provides a more comprehensive assessment of how lifestyle factors intersect with PCOS symptomatology.

## Materials and methods

### Study design and participants

This web-based analytical cross-sectional study was conducted between April and July 2024 among women aged 18–45 years living in Türkiye who reported having received a physician diagnosis of polycystic ovary syndrome (PCOS). Participants were recruited using a convenience sampling strategy through PCOS-related online platforms, including social media support groups and forums. Eligibility criteria included Turkish language proficiency, provision of informed consent, and self-report of a PCOS diagnosis accompanied by clinical details such as year of diagnosis and type of healthcare facility. Women who were pregnant or lactating, had a self-reported history of psychiatric or eating disorders, were receiving medical nutrition therapy, reported chronic conditions such as diabetes mellitus or cardiovascular disease, or were using medications known to substantially affect metabolic status (e.g., hormonal contraceptives or metformin) were excluded. Because PCOS diagnosis could not be clinically verified within the context of an online survey, phenotypic classification and diagnostic criteria (e.g., Rotterdam criteria) could not be confirmed. Diagnostic details were therefore collected to enhance transparency regarding self-reported PCOS status.

### Sample size

The required sample size was determined *a priori* using G*Power 3.1 based on planned primary correlation analyses examining associations among nutrition knowledge, symptom burden, and QoL outcomes. Assuming a small-to-moderate effect size (*r* = 0.20), a two-tailed *α* = 0.05, and statistical power of 80%, the minimum recommended sample size was estimated to be 194 participants. Of the 403 individuals who completed the survey, 330 met the inclusion criteria and were included in the final analysis.

### Ethical considerations

Ethical approval for the study was obtained from the Non-Interventional Clinical Research Ethics Committee of Fenerbahçe University (12 March 2024, Protocol No, 41.2024fbu). All procedures were performed in accordance with the principles of the Declaration of Helsinki, and informed consent was obtained electronically from all participants prior to data collection. The study protocol complied with relevant institutional and national guidelines.

### Data collection tools

#### Sociodemographic and clinical characteristics

Participants provided self-reported data on age, educational attainment, marital and employment status, height, weight, duration of PCOS diagnosis, primary sources of PCOS-related information, and the regularity and frequency of physical activity. Body mass index (BMI) was calculated as weight in kilograms divided by height in meters squared (kg/m^2^). BMI categories were defined according to the World Health Organization classification as underweight (<18.5 kg/m^2^), normal weight (18.5–24.9 kg/m^2^), overweight (25.0–29.9 kg/m^2^), and obese (≥30.0 kg/m^2^) (24).

#### Assessment of PCOS symptoms

PCOS-related symptoms were assessed using a seven-item structured symptom checklist designed to evaluate self-perceived symptom frequency over the past 3 months. The checklist was developed specifically for this study to provide a concise assessment of commonly reported PCOS symptoms in an online survey setting. Based on two recent authoritative reviews (25, 26), seven symptom domains commonly reported in women with PCOS were identified and included in the checklist: menstrual irregularities, hirsutism, acne, insulin-resistance-related symptoms (e.g., increased appetite, sugar cravings, and postprandial fatigue), skin pigmentation, weight gain or difficulty losing weight, and hair loss. The selected items reflected symptoms frequently reported in the literature and considered clinically relevant to the assessment of PCOS symptom burden. Each item was rated on a 5-point frequency scale, ranging from 0 (“never”) to 4 (“always”). Item scores were summed to generate a total symptom burden index ranging from 0 to 28, with higher scores reflecting a greater overall symptom severity. Because the items represent distinct clinical manifestations rather than a unified latent construct, the total score was treated as an index measure, rather than a psychometric scale. This indexing approach is consistent with PRO-based symptom burden assessments in previous research, for which internal consistency is not conceptually appropriate for heterogeneous symptom clusters (27).

#### Nutrition knowledge assessment

Nutrition knowledge was assessed using the Nutrition Knowledge Level Scale for Adults (NKLSA), developed and validated for the Turkish population by Batmaz (28). This reliable and valid tool comprises 32 items divided into two subscales: basic nutrition knowledge (20 items) and food preferences (12 items). Each item is rated on a 5-point Likert scale, where higher scores are assigned to “strongly agree” responses for correct statements and to “strongly disagree” responses for incorrect ones. The maximum achievable score is 80 for the basic nutrition subscale and 48 for the food preferences subscale. The reliability coefficients (Cronbach’s alpha) were 0.72 for the basic nutrition subscale and 0.70 for the food preferences subscale.

#### HRQoL assessment

Participants’ QoL was assessed using the Short Form-12 Health Survey (SF-12), originally developed by Ware et al. (29) and validated in Turkish by Soylu and Kütük (30). The scale includes 12 items covering eight dimensions of physical and mental health. Two summary scores were derived: the Physical Component Summary (PCS-12) and the Mental Component Summary (MCS-12). Higher scores indicate better perceived health status. Internal consistency values were 0.72 for PCS-12 and 0.73 for MCS-12.

### Statistical analysis

All statistical analyses were conducted using the Statistical Package for the Social Sciences (SPSS) version 26.0 (IBM Corp., Armonk, NY, United States). Continuous variables were presented as mean ± standard deviation, and categorical variables were summarized as frequencies and percentages. The normality of continuous variables was assessed using the Shapiro–Wilk test and visual inspection of histograms and Q–Q plots. For group comparisons, independent samples *t*-tests and one-way analysis of variance (ANOVA) were used for normally distributed variables, while the Mann–Whitney *U* test and Kruskal–Wallis test were applied when normality assumptions were not met. When ANOVA results were significant, post-hoc comparisons were performed using Tukey’s Honestly Significant Difference (HSD) test for equal variances or Bonferroni-adjusted tests as appropriate. Associations between continuous variables were examined using Pearson correlation coefficients. Dichotomous variables, such as regular exercise status, were not included in correlation analyses and were instead evaluated using appropriate group comparison tests. To identify independent predictors of total PCOS symptom burden, a multiple linear regression analysis was performed including age, BMI, education level, physical activity status, duration of PCOS diagnosis, nutrition knowledge score, food preference score, PCS-12, and MCS-12 as independent variables. Model assumptions were assessed by examining the normality of residuals, multicollinearity (variance inflation factor), and homoscedasticity. A two-tailed *p*-value of <0.05 was considered statistically significant.

## Results

The sociodemographic and clinical characteristics of the participants are presented in Table 1. This study included 330 women with PCOS, with a mean age of 27.23 ± 4.8 years and a mean BMI of 26.15 ± 5.8 kg/m^2^. Most participants held a university or postgraduate degree (96.4% combined), were single (61.5%), and were employed or students (80.3%). Regarding clinical characteristics, 70.9% reported not engaging in regular physical activity. The duration of PCOS diagnosis varied, with 31.8% reporting 2–5 years and 27.9% reporting 6–10 years since diagnosis. Based on BMI categories, 47.6% were normal weight, 28.2% overweight, and 22.1% obese. The mean duration of PCOS diagnosis was 7.27 ± 5.9 years (Table 1).

**Table 1:** Sociodemographic and clinical characteristics of the participants (*n* = 330).

Category	Frequency (*n*)	Percentage (%)
Age (mean ± SD)	27.23 ± 4.8	
Weight (kg) (mean ± SD)	69.73 ± 16.3	
BMI (kg/m^2^) (mean ± SD)	26.15 ± 5.8	
PCOS diagnosis duration (mean ± SD)	7.27 ± 5.9	
Education level		
High school or below	12	3.6
University	197	59.7
Postgraduate and above	121	36.7
Marital status		
Married	127	38.5
Single	203	61.5
Employment status		
Employed	178	53.9
Unemployed	56	17.0
Student	87	26.4
Housewife	9	2.7
PCOS diagnosis duration		
0-1 year	55	16.7
2–5 year	105	31.8
6–10 year	92	27.9
11 year and above	78	23.6
Regular exercise habit		
Yes	96	29.1
No	234	70.9
BMI classification (kg/m^2^)		
Underweight (<18.5)	7	2.1
Normal (18.5–24.9)	157	47.6
Overweight (25–29.9)	93	28.2
Obese (30–39.9)	73	22.1

Comparisons of SF-12 and NKLSA scores across education level, BMI classification, exercise habits, and PCOS diagnosis duration are presented in Table 2. BMI categories were significantly associated with physical health status, with overweight and obese participants showing lower PCS-12 scores than those with normal weight (*p* = 0.013). Participants reporting regular physical activity had significantly higher PCS-12 scores compared to those who were inactive (*p* = 0.002), while no significant differences were observed for other variables. In contrast, MCS-12, nutrition knowledge, food preference scores, and total NKLSA scores did not differ across education levels, BMI categories, exercise habits, or PCOS diagnosis durations (all *p* > 0.05). Total PCOS symptom burden varied only by BMI classification, with overweight and obese women reporting higher symptom scores than their normal-weight counterparts (*p* < 0.001) (Table 2).

**Table 2:** Comparison of SF-12 and NKLSA scores across education levels, BMI categories, exercise habits, and PCOS symptom burdens.

Category	PCS-12	MCS-12	Nutrition knowledge	Food preferences	PCOS symptom score
Education level					
High school or below	43.41 ± 7.21	39.11 ± 5.32	55.00 ± 6.59	42.42 ± 4.81	18.00 ± 8.71
University	43.99 ± 5.91	36.45 ± 5.83	52.91 ± 6.60	39.21 ± 5.37	16.23 ± 6.52
Postgraduate and above	43.61 ± 5.56	36.37 ± 5.93	52.45 ± 7.06	38.63 ± 6.06	15.49 ± 5.92
*p*-value	0.827	0.270	0.440	0.089*	0.272
BMI classification					
Underweight	44.54 ± 6.02^a,b^	35.15 ± 6.91	51.57 ± 4.12	39.86 ± 6.52	14.29 ± 8.26^a^
Normal	44.87 ± 5.72^a^	36.25 ± 6.11	52.52 ± 6.78	39.03 ± 5.80	15.87 ± 6.78^a^
Overweight	43.10 ± 5.61^a,b^	37.28 ± 5.80	53.08 ± 6.70	38.48 ± 5.76	19.94 ± 6.22^b^
Obese	42.46 ± 5.99^b^	36.24 ± 5.26	53.25 ± 7.10	40.03 ± 5.04	20.45 ± 6.87^b^
*p*-value	**0.013**	0.430	0.808	0.405	<0.001
Regular exercise habit					
Yes	45.41 ± 5.06	35.88 ± 5.63	53.39 ± 6.86	38.80 ± 6.20	17.22 ± 6.78
No	43.18 ± 5.99	36.78 ± 5.94	52.58 ± 6.73	39.24 ± 5.41	18.31 ± 7.08
*p*-value	0.002**	0.216**	0.328**	0.726**	0.151**
PCOS diagnosis duration					
0-1 year	42.34 ± 5.93	36.75 ± 5.30	53.76 ± 6.10	39.96 ± 5.15	16.48 ± 7.53
2–5 year	44.84 ± 5.49	36.15 ± 5.98	52.57 ± 7.64	39.63 ± 5.89	18.08 ± 6.75
6–10 year	43.58 ± 5.91	37.19 ± 6.99	52.33 ± 5.57	37.93 ± 5.17	17.77 ± 6.74
11 year and above	43.70 ± 6.00	36.43 ± 5.44	52.85 ± 7.21	39.10 ± 6.01	19.18 ± 7.18
*p*-value	0.075	0.669	0.647	0.106	0.181

Values are presented as mean ± standard deviation. One-way analysis of variance, *Kruskal–Wallis test, **Independent samples *t*-test. Different superscript letters (a, b) within the same column indicate statistically significant differences between groups; *p* ≤ 0.05. Bold values indicate statistically significant results (*p* < 0.05).

Correlations between PCOS-related symptoms and demographic, clinical, nutrition, and quality-of-life variables are presented in Table 3. BMI demonstrated consistent positive correlations with multiple PCOS-related symptoms, including hirsutism (*r* = 0.183), insulin-resistance symptoms (*r* = 0.471), skin pigmentation (*r* = 0.180), weight gain (*r* = 0.586), and hair loss (*r* = 0.148). PCOS diagnosis duration was positively associated with hirsutism (*r* = 0.139), insulin-resistance symptoms (*r* = 0.123), and skin pigmentation (*r* = 0.137). PCS-12 scores were negatively associated with several symptoms, including menstrual irregularities (*r* = −0.142), insulin-resistance symptoms (*r* = −0.201), and weight gain (*r* = −0.233). MCS-12 scores were only associated with weight gain (*r* = −0.113). No significant correlations were observed between nutrition knowledge, food preference scores, or educational level and PCOS-related symptoms (Table 3).

**Table 3:** Correlations between PCOS-related symptoms and demographic, clinical, lifestyle, nutrition, and quality-of-life variables.

Variable	Menstrual irregularity	Hirsutism	Acne	Insulin resistance	Skin pigmentation	Weight gain	Hair loss
Age	−0.053	−0.044	−0.097	0.047	0.018	−0.027	0.031
BMI	0.101	0.183*	−0.016	0.471**	0.180*	0.586**	0.148*
Education level	−0.015	−0.027	−0.027	−0.015	−0.030	−0.002	−0.016
PCOS diagnosis duration	0.106	0.139*	−0.054	0.123*	0.059	0.137*	0.056
Nutrition knowledge score	0.014	0.022	−0.036	−0.034	0.021	−0.026	0.027
Food preferences score	0.031	0.034	−0.042	−0.016	0.006	0.022	0.057
PCS-12	−0.142*	−0.088	−0.073	−0.201**	−0.067	−0.233**	−0.095
MCS-12	−0.061	−0.048	−0.087	−0.066	−0.059	−0.113*	−0.033

Values represent Pearson correlation coefficients (*r*). *p < 0.05, **p < 0.001.

The results of the multiple linear regression analysis predicting total PCOS symptom burden are presented in Table 4. BMI was the strongest positive predictor of total PCOS symptom burden (*β* = 0.309, *p* < 0.001), indicating higher symptom scores among women with greater BMI. PCS-12 scores were inversely associated with symptom severity (*β* = −0.211, *p* = 0.002), suggesting that poorer physical health status was associated with higher symptom burden. No significant associations were observed for age, education level, regular exercise, diagnosis duration, nutrition knowledge, food preferences, or MCS-12 scores (*p* > 0.05). The model explained 24.6% of the variance in total symptom scores (Adjusted *R*^2^ = 0.214; model *p* < 0.001) (Table 4).

**Table 4:** Multiple linear regression model predicting total PCOS symptom score.

Predictor	*β*	SE	95% CI	*p*
Age	−0.011	0.066	−0.141, 0.119	0.869
BMI	**0.309**	0.080	0.151, 0.466	**<0.001**
Education level	−0.302	0.792	−1.863, 1.260	0.704
Regular exercise	1.082	0.987	−0.857, 3.022	0.273
PCOS diagnosis duration	−0.038	0.100	−0.235, 0.158	0.709
Nutrition knowledge score	−0.012	0.051	−0.112, 0.089	0.811
Food preferences score	−0.028	0.072	−0.169, 0.113	0.701
PCS-12	**−0.211**	0.067	−0.343, −0.079	**0.002**
MCS-12	−0.097	0.061	−0.217, 0.022	0.110
Model fit	*R*^2^ = 0.246; Adjusted *R*^2^ = 0.214; Model *p* < 0.001			

Bold values indicate statistically significant results (*p* < 0.05).

## Discussion

Polycystic ovary syndrome is a chronic endocrine disorder affecting both metabolic and reproductive health and is strongly influenced by lifestyle factors (31). Using an integrated analytical framework, this study examined the associations between nutrition knowledge, diet-related preferences, symptom burden, and HRQoL in women with PCOS. Although BMI was strongly associated with both physical functioning and symptom burden, physical activity was mainly associated with physical functioning rather than overall symptom burden. The findings further highlight that higher nutrition knowledge was not accompanied by better clinical outcomes. Overall, these results suggest that lifestyle behaviors and weight status may be more closely linked to PCOS-related outcomes, while nutrition knowledge alone may be insufficient to influence health outcomes without accompanying behavioral and contextual factors.

In this study, participants had a mean BMI of 26.15 kg/m^2^, with approximately half classified as normal weight and the remaining participants classified as overweight or obese. The observation of lower PCS-12 scores and higher symptom burden among women with higher BMI indicates that body weight status is strongly associated with both physical functioning and symptom burden in PCOS. This finding is consistent with recent studies reporting that elevated BMI is linked to poorer quality of life, both directly and indirectly through its associations with body image concerns and eating-related behaviors (32). Increased body weight, often accompanied by insulin resistance, hyper-androgenism, and low-grade inflammation, has been associated with greater severity of menstrual irregularities, difficulties in weight management, hirsutism, and fatigue (17, 21). In line with these observations, BMI showed the strongest association with total symptom burden in the multivariable model, highlighting its relevance to PCOS symptom expression.

Regular physical activity was positively associated with PCS-12 scores, underscoring the relevance of physical activity for physical health status in women with PCOS. Previous evidence suggests that physical activity is associated with improvements in metabolic and reproductive health through reductions in insulin resistance and central adiposity, as well as support for menstrual regulation, thereby contributing to lower cardio-metabolic risk (33, 34). However, no independent association was observed between physical activity and overall symptom burden in multivariable analyses. This finding suggests that the relationship between physical activity and symptom burden may be influenced by other interrelated lifestyle and metabolic factors. Given the cross-sectional design, these associations should be interpreted with caution.

In this study, nutrition knowledge and diet-related preference scores did not differ significantly according to education level, BMI category, physical activity habits, or duration since PCOS diagnosis, nor were they associated with health-related quality of life or symptom burden. These findings align with those of Spronk et al. (18), who reported that associations between nutrition knowledge and dietary intake in adults are generally positive but weak, suggesting that nutrition knowledge alone has a limited and inconsistent association with health behaviors and clinical outcomes. Similarly, a recent study examining nutrition literacy and HRQoL in adults identified only a very weak positive correlation and no significant differences across most sociodemographic or lifestyle variables (35). Taken together, this body of evidence supports the interpretation that knowledge-related constructs, while necessary, may be insufficient on their own to influence health-related outcomes without accompanying motivational, behavioral, or contextual factors.

From a behavioral perspective, these findings can be interpreted within the Capability, Opportunity, Motivation–Behavior (COM-B) framework, which conceptualizes behavior as the result of interactions between capability, opportunity, and motivation (36). Within this framework, nutrition knowledge may represent psychological capability; however, capability alone is unlikely to lead to sustained behavioral change in women with PCOS. Previous research has demonstrated that effective lifestyle modification in PCOS depends not only on knowledge but also on motivational and environmental factors that enable or constrain behavior, including psychological and environmental barriers as well as limited motivational and social support (37, 38). This perspective may help explain why, in the present study, higher nutrition knowledge was associated with more favorable diet-related preferences but was not reflected in improved symptom burden or HRQoL outcomes. This finding suggests that nutrition knowledge alone may be insufficient to produce meaningful health improvements without additional motivational and behavioral support.

Within the context of PCOS, previous research has shown that emotional eating, binge eating, body dissatisfaction, and an elevated risk of eating disorders are common and may interfere with the adoption of healthier dietary patterns (15, 39). A recent review further reported that eating disorders in women with PCOS frequently co-occur with depression, anxiety, and low self-esteem and are often accompanied by obesity and negative body image, collectively complicating weight management and long-term quality of life (40). In this context, the absence of an association between nutrition knowledge and health-related quality of life or symptom burden in the present study—despite partial alignment of higher knowledge with healthier diet-related preferences—suggests a disconnect between knowledge and health outcomes rather than a directly measured behavioral gap. Because dietary intake and eating behaviors were not directly assessed, this finding should be interpreted as indicating that nutrition knowledge alone may be insufficient to influence clinical outcomes without additional psychological, motivational, or contextual support.

Regarding HRQoL, PCS-12 scores decreased significantly with increasing menstrual irregularity, insulin resistance–related symptoms, and perceived weight gain, whereas MCS-12 scores were associated only with weight gain. Among the evaluated symptoms, perceived weight gain showed the strongest negative association with physical HRQoL, followed by insulin resistance–related symptoms and menstrual irregularity. This pattern suggests that symptom burden in PCOS more strongly affects physical aspects of HRQoL, while mental well-being may be more selectively influenced by weight- and appearance-related concerns. Consistent with this interpretation, previous studies have shown that weight status, hirsutism, and acne are associated with higher levels of depression, anxiety, and body image distress, ultimately contributing to reduced HRQoL in women with PCOS (41–43). In line with these findings, the present results indicate greater impairment in physical HRQoL among women reporting prominent weight gain and insulin resistance–related symptoms.

Overall, these findings indicate that improving nutrition knowledge alone may be insufficient to enhance health-related outcomes in PCOS, underscoring the need for more comprehensive, behavior-oriented approaches to support sustainable lifestyle change. In line with this interpretation, Arentz et al. (44) reported that although many women with PCOS attempt dietary or physical activity modifications, only a minority experience meaningful benefits, often due to psychosocial, motivational, and practical barriers. Accordingly, strategies that incorporate psychosocial support, motivational interviewing, self-monitoring, and goal setting may be particularly relevant for facilitating sustained behavior change. Weight management and regular physical activity remain closely associated with lower symptom burden and better physical HRQoL in women with PCOS. Evidence from randomized controlled trials suggests that lifestyle interventions resulting in weight reduction are associated with improvements in insulin resistance, glycemic markers, and menstrual regularity (8, 9). Given the interconnected metabolic, reproductive, and psychological dimensions of PCOS, multidisciplinary lifestyle interventions are increasingly emphasized, with integrated care models involving dietitians, endocrinologists, gynecologists, and mental health professionals shown to support lifestyle adherence and symptom management.

Strengths of this study include an adequately powered sample size determined through *a priori* power analysis, the use of validated and reliable instruments, and the simultaneous examination of nutrition knowledge, diet-related preferences, symptom burden, and health-related quality of life within a single analytical framework. The development of a PCOS-specific symptom burden index enabled a comprehensive assessment of symptom severity across multiple domains, while acknowledging its role as a descriptive summary measure rather than a diagnostic tool. Several limitations should be considered. The cross-sectional design precludes causal inference, and reliance on self-reported PCOS diagnosis and anthropometric data may introduce misclassification or reporting bias. In addition, dietary behavior was assessed at the level of diet-related preferences rather than actual dietary intake, nutrient consumption, or diet quality indicators. Therefore, conclusions regarding nutrition-related behaviors should be interpreted with caution. The exclusion of women using pharmacological treatments for PCOS and the relatively high educational attainment of the sample may further limit the generalizability of the findings to more socioeconomically diverse or clinically treated populations.

Nevertheless, the consistency of findings across multiple analytical models and the relatively large sample size support the relevance of the results for informing public health and women’s health planning. Future research should employ longitudinal designs, incorporate objective dietary, anthropometric, and biochemical measures, and integrate psychosocial and behavioral determinants—such as emotional eating, body image, motivation, self-efficacy, and dietary adherence—to better elucidate pathways linking nutrition knowledge, behavior, and health outcomes in women with PCOS.

## Conclusion

This study suggests that nutrition knowledge alone is not independently associated with symptom burden or health-related quality of life in women with PCOS. In contrast, body mass index and physical activity were more consistently associated with symptom severity and physical functioning. These findings indicate that information-based approaches on their own may be insufficient to address PCOS-related health outcomes and highlight the importance of comprehensive, behavior-oriented lifestyle strategies. Integrating nutrition education with psychosocial support, motivational counseling, weight management, and physical activity promotion may better support sustained behavior change, improve symptom management, and contribute to more meaningful improvements in quality of life among women with PCOS.

## Data Availability

The raw data supporting the conclusions of this article will be made available by the authors, without undue reservation.
